# Isolation of bacteria from fermented food and grass carp intestine and their efficiencies in improving nutrient value of soybean meal in solid state fermentation

**DOI:** 10.1186/s40104-018-0245-1

**Published:** 2018-04-05

**Authors:** Samantha Medeiros, Jingjing Xie, Paul W. Dyce, Hugh Y. Cai, Kees DeLange, Hongfu Zhang, Julang Li

**Affiliations:** 1 Department of Animal BioSciences, University of Guelph, Guelph, Canada; 2 State Key Laboratory of Animal Nutrition, Institute of Animal Sciences, Chinese Academy of Agricultural Sciences, Beijing, 100193 China; 3 Animal Science Department, Auburn University, Auburn, AL 36849 USA; 4 Animal Health Laboratory, University of Guelph, Guelph, Canada; 5 College of Life Science and Engineering, Foshan University, Foshan, Guangdong China

**Keywords:** Allergens, Fermentation, Food source bacteria, Soybean meal

## Abstract

**Background:**

Soybean meal is an excellent and cost-effective protein source; however, its usage is limited in the piglet due to the presence of anti-nutritional factors and the antigens glycinin and β-conglycinin. The objective of the current study was to screen and select for bacteria that can be efficiently adopted to ferment soybean meal in order to solve this problem.

**Results:**

Bacteria were isolated from fermented soy foods and the grass carp intestine, and strains selected for high protease, cellulase and amylase activities. The isolated bacteria were characterized as *Bacillus cereus, Bacillus subtilis and Bacilus amyloliquefacien,* respectively. Fermentation with food-derived Isolate-2 and fish-derived F-9 increased crude protein content by 5.32% and 8.27%, respectively; improved the amino acid profile by increasing certain essential amino acids, broke down larger soy protein to 35 kDa and under, eliminated antigenicity against glycinin and β-conglycinin, and removed raffinose and stachyose in the soybean meal following a 24-h fermentation.

**Conclusions:**

Our results suggest these two *B. amyloliquefaciens* bacteria can efficiently solid state ferment soybean meal and ultimately produce a more utilizable food source for growing healthy piglets.

## Background

Weaning is a critical period during swine production as sucking piglets are separated from the sow and transitioned to solid feed. The industrial prevalence of early weaning at 21–26 d of age resulted in reduced piglet performance including decreased feed intake, diarrhea, and retarded growth, due to many factors including an immature gastrointestinal system [1, 2]. Along with the underdeveloped immune function [2], separation stress [3] and dietary transition are required at the time of weaning. Due to the newly weaned pig facing these challenges their diets generally consist of highly digestible and palatable animal-based proteins such as: dried whey, spray dried plasma, poultry by-product meal and fish meal. There has been a push to try to limit these animal proteins due to possible risk of pathogen spread and rising costs. Soybean meal would be a more cost-effective alternative due to its high protein content, excellent amino acid profiles as well as the positive isoflavone properties [4–7].

Despite the high nutritional values soybean meal also has a few anti-nutritional factors, including glycinin and β-conglycinin, non-starch polysaccharides (NSP), raffinose family oligosaccharides (RFOs), trypsin inhibitors, and lectin [8–10]. Due to their underdeveloped digestive and immune functions young animals at weaning stage are less tolerant to these factors than more developed finisher pigs. Some heat labile factors such as trypsin inhibitors and lectin in the soybean can be deactivated by heat during feed processing [8, 9]. However, other factors, such as the antigenic proteins glycinin and β-conglycinin, can still have negative effects on the piglet. Glycinin has been well known to damage intestine morphology, interfere with the immune system, and increase diarrhea resulting in decreased growth performance in piglets [11–15]. β-conglycinin can elicit a hypersensitive immune response and negatively affect piglet growth performance [13, 16, 17]. Indigestible RFOs and NSP can result in discomfort in the gastrointestinal tract, changes in the digesta viscosity, and impede nutrient digestibility, ultimately negatively impacting growth performance in young piglets [10, 18, 19].

Microbial fermentation is an effective and cheap means to overcome the issues of soybean meal. Previous studies using molds, such as the *Aspergillus* species for soybean meal fermentation degraded the large antigenic proteins, increased the crude protein contents, and improved the non-essential amino acid profile [20, 21]. Feeding trials in piglets demonstrated the improved feed efficiency, amino acid digestibility and blood urea nitrogen when 10–15% fermented soybean meal was included in the diet [22]. It has also been shown that the standardized ileal digestibility of amino acids in fermented soybean meal were similar to that of fish meal [23]. These studies provide supports for using fermented soybean meal in the diet of early-weaned pigs. Besides the *Aspergillus* species, bacteria such as *Bacillus* and *Lactobacillus* species are also used for the fermentation of soybean meal [24]. Compared with mold fermentation using bacteria could increase protein solubility, digestibility and the percentages of small-sized peptides in the soybean meal [24]. The function of different types of microorganisms in the fermentation depends on their extracellular enzymes, including proteases, amylases, and α-galactosidases [25], and therefore the choice of the organism for fermentation may have profound effects on the nutritional quality of the final fermented soybean meal.

Traditional Chinese fermented soy foods undergo natural spontaneous fermentation [26] and contain various bacteria with high enzyme activities. The primarily herbivorous grass carp feeds on aquatic plants and has been found to contain bacteria with cellulolytic properties [27–30]. The fermented soy products and the grass carp are two desirable sources for isolating generally recognized as safe bacteria with high protease and cellulolytic properties in order to degrade soy antigens and structural carbohydrates of the soybean meal. The current study aimed to identify new food- and intestine-derived bacteria which are good candidates for soybean meal fermentation to improve the nutrition values.

## Methods

### Media preparation

Luria Bertani (LB), Bacto Brain Heart Infusion (BHI), and DeMan, Rogosa and Sharpe (MRS) agar plates were used to isolate bacteria colonies. LB agar (pH 7.5) were prepared with 1.0 g of tryptone powder (Bio Basics Canada INC, Canada), 0.5 g of yeast extract (Bio Basics Canada INC, Canada), 1.0 g of NaCl (Fisher Scientific, USA) and 1.5 g of BactoAgar in 100 mL deionized water. BHI (Becton, Dickinson & Company, USA) and MRS (Oxoid, England) agar plates were prepared according to manufacturer’s procedures with the addition of 1% (*w*/*v*) BactoAgar.

Soy milk, carboxymethyl cellulose (CMC), starch agar plates were used to select colonies with proteolytic, cellulolytic and amylase activities, respectively. Soy milk agar was made by mixing 1 g of soy milk powder (Bulk Barn, Canada) and 2.5 g of BactoAgar (Becton, Dickinson & Company, USA) with 100 mL of water as described by Amoa-Awua et al. [31]. CMC agar plates were prepared by adding 1.0 g of CMC (Acros Organics, USA) and 1.5 g of BactoAgar to 100 mL of deionized water as previously described by Sazci et al. [32]. To prepare starch agar plates, 1 g of corn starch (Bulk Barn, Canada) and 1.5 g of BactoAgar were added to 100 mL of deionized. All plates were autoclaved for 25 min at 121 °C.

### Bacteria isolation and screening for protease, cellulase and amylase activities

Five types of fermented soy products, including black fermented beans, red fermented beans, yellow fermented beans, soft fermented beans and Japanese Natto, were obtained from the local supermarket and used to isolate proteolytic bacteria. Approximately 1 cm^3^ of each source of fermented products was mixed with 500 μL of sterile water and serial dilutions were conducted to isolate approximately 100 clearly defined individual colonies of bacteria per plate. A hundred microliter of diluted samples from each of the five sources was plated onto the soymilk agar and incubated for 24 h in 37 °C. For each food source, colonies demonstrating the largest clear zone were selected and glycerol stocks were created for storage of isolates at − 80 °C. A total of six isolates were subjected to further screening for cellulase and amylase activity.

Contents from the proximal, middle and distal section of grass carp intestine were harvested to isolate bacteria with cellulolytic activity [30]. Diluted samples were first plated onto LB, BHI and MRS agar. Any colony present on the three plates was blotted onto the soymilk and CMC plates and incubated at 37 °C for 72 h. The clearance zone in the CMC agar plates was revealed by Congo Red staining as described [32, 33]. Colonies which were able to grow on the soymilk agar and exhibited the highest cellulase activity were selected and a total of three colonies were selected and further screened for enzymatic activities.

A single colony of each selected isolate was inoculated into 1 mL of BHI culture media and incubated for 18 h at 37 °C to extract crude enzymes [34]. The supernatant was harvested by centrifuging the liquid culture at 10,000 r/min for 15 min at 4 °C using an Eppendorf 5415D centrifuge. Twenty microliters of each crude enzyme extract were pipetted into the punctured hole on the soy milk, CMC and starch agar plates, respectively, and incubated at 37 °C for 72 h. Enzymatic activities were ranked by the clearance zone measurements taken from the edge of the colony (soy milk agar plate) or the edge of punctured hole (CMC and starch agar plates) to the outer edge of the clearance zone. The amylase clearance zone was revealed using an additional Gram’s Iodine Stain [31]. Each experiment was repeated three times using fresh inoculums each time. Commercial *B. subtilis* ATCC 6633 with protease [35], amylase [36] and cellulase activities [37] was used as the positive control and the blank culture media was used as the negative control.

### Bacteria identification by MALDI-TOF MS

All selected bacteria selected were characterized to the species or genus level by matrix assisted laser desorption ionization- time of flight mass spectrometry (MALDI-TOF MS). Isolates were individually streaked onto the BHI agar plate, and incubated for 18 h at 37 °C. Each fresh cultured bacterial colony was spotted onto the target plate (Bruker), air dried, and overlaid with 1 μL matrix solution mixed with an organic solvent solution made of 50% acetonitrile (Sigma Aldrich, Canada) and 2.5% trifluoroacetic acid (VWR, Canada). The target plate was then placed onto the MALDI-TOF MS instrument (Bruker) for analysis. Peptide mass fingerprint spectra was scored using MALDI-TOF MS software and database (MALDI Biotyper 3.0, Bruker). An isolate was automatically identified to the species level if the score was 2.0 to 3.0, or to genus level if the score was 1.7 to 1.999. No reliable identification was generated if the score was less than 1.7.

### Sequencing analysis of 16S rRNA and *gyrB* genes

The 16S rRNA and *gyrB* genes were used to further identify the strain of bacteria isolates. Genomic DNA of each isolate was extracted using the PureLink Genomic DNA Mini Kit (Invitrogen, USA) according to manufacturers’ instructions. The partial 16S rRNA gene was amplified and sequenced using the same primer set of BSF8/20 and BSR534/18 [38] and the *gyrB* gene was amplified by the universal primer set of UP-1 and UP-2r and sequenced by the primer set of UP1S and UP2SR (Table 1) [39]. Before DNA sequencing using ABI Prism 3100 Automated Sequencer, the size of each PCR product was verified by agarose gel electrophoresis and the concentration of the PCR products was determined using a BioPhotometer Plus spectrometer (Eppendorf, USA) to ensure that the minimum concentration required. Bacterial strains were identified by blasting the sequencing results from both genes against nucleotide sequences in the GeneBank database (http://blast.ncbi.nlm.nih.gov/Blast.cgi).

**Table 1:** Amplification and sequencing primers for the 16S rRNA and *gyrB* genes

Gene	Primer	Primer sequence (5′→3′)
16S rRNA	BSF8/20 (Forward)	AGAGTTTGATCCTGGCTCAG
	BSR534/18 (Reverse)	ATTACCGCGGCTGCTGGC
*gyrB*	UP-1 (Forward)	GAAGTCATCATGACCGTTCTGCA(TC)GC(TCAG)GG(TCAG)GG(TCAG)AA(AG)TT(TC)GAGAAGTCATCATGACCGTTCTGCAYGCNGGNGGNAARTTYGA
	UP-2r (Reverse)	AGCAGGGTACGGATGTGCGAGCC(AG)TC(TCAG)AC(AG)TC(TCAG)GC(AG)TC(TCAG)GTCATAGCAGGGTACGGATGTGCGAGCCRTCNACRTCNGCRTCNGTCAT
	UP1S (Forward)	GAAGTCATCATGACCGTTCTGCA
	UP2SR (Reverse)	AGCAGGGTACGGATGTGCGAGCC

### Solid state fermentation of soybean meal

Soybean meal (48% crude protein content, Grand Valley Fortifiers, Canada) was sterilized by mild heating at 125 °C for 30 min to minimize lysine loss [40]. For each fermentation, bacteria inoculums of selected isolated were prepared fresh by incubating a single colony in the BHI culture media for 18 h at 37 °C to obtain a culture of 10^8^ colonies forming unit (CFU)/mL. Solid state fermentation was set up by inoculating 2 × 10^8^ CFU/mL isolated bacteria into the 2 g of soybean meal for each of the treatment group with a volume: weight ratio of 2:1. This mixture was set to ferment in an incubator at 42 °C for 48 h without any agitation. Each fermentation was performed in triplicate. Samples were collected at 24 and 48 h and stored at − 80 °C. Lyophilized samples were then ground into a fine powder for further analyses.

### Determination of soluble protein fractions and distribution

Total soluble proteins of the unfermented and fermented soybean meals were isolated as previously described by Hong et al. [20]. The quantification was carried out using the DC Assay Kit (Bio-Rad Laboratories, USA). Fractions of soluble protein extract were analyzed by 11% tris sodium–dodecyl sulfate-polyacrylamide gel electrophoresis (SDS-PAGE). A volume of 15 μL of denatured total soluble protein sample was loaded to the gel, and subjected to electrophoresis for 100 min using 100 V. The gel was stained with Coomassie blue. Images were taken using ChemiDoc XRS+ (Bio-Rad Laboratories, USA) and analyzed with the Image Lab software.

### In vitro determination of pig plasma immunoreactivity

To determine piglets’ immunoreactivity against an unfermented and fermented soybean meal, Western blots were performed. Total soluble protein of 50 μg each was loaded into an 11% SDS-PAGE as described above and then transferred to an Immobolin-P (Millipore, Billerica, USA) polyvinyl difluoride transfer membrane using the wet transfer method at 100 V for 100 min. The membrane was first blocked with buffer comprised of 5% skim milk, 0.1% Tween 20, and 1× TBS for 1 h before the primary antibody incubation. After several washes with washing buffer (0.1% Tween and TBS), the membrane was incubated with the primary antibody at 4 °C overnight. The primary antibody was prepared against soybean antigenic proteins glycinin and β-conglycinin following a published protocol [41]. Briefly, plasma was collected from 8-week-old piglets that were previously exposed to feed containing soybean meal for 21 d and developed an immune response to soybean allergens. The primary antibody was comprised of 20 μL of pooled pig serum and diluted to 1:500 with the blocking buffer. Rabbit anti-pig horseradish peroxidase-linked IgE was used as the secondary antibody (1: 100,000; Abcam). Proteins were detected by using Clarity Western Enhanced Chemiluminescence substrate (Bio-Rad Laboratories, USA) according to manufacturer’s instructions.

### Proximate analysis and amino acid profiling, oligosaccharide concentrations

Lyophilized samples of unfermented and fermented soybean meal were subjected to the measurement of crude protein and amino acid profiles according to the corresponding AOAC official methods (moisture, method 934.01; crude protein, method 954.01; amino acids, method 994.12). Raffinose and stachyose were determined using high-performance liquid chromatography (HPLC, Shimadzu LC-15C, Japan) equipped with a differential detector (Shimadzu RID-10A, Japan) as described by Yao et al. [42]. Briefly, stachyose and raffinose were extracted by 70% (*v*/*v*) ethanol with the assistance of microwave. The supernatant was concentrated and reconstituted to 25 mL. A volume of 20 μL of each sample was injected into the HPLC system with 70% acetonitrile as the mobile phase. The temperature was set at 35 °C, and the flow rate was controlled at 1 mL/min. Serial diluted standards of stachyose (Dr. Ehrenstorfer, Germany) and raffinose (Dr. Ehrenstorfer, Germany) were used to determine the stachyose and raffinose concentrations.

### Statistical analysis

All statistical analyses were performed using PRISM GraphPad Prism Version 5.03 (San Diego, CA). The data for bacteria screening were analyzed using one-way ANOVA with a Dunnet post-test to determine if any treatment group was significantly different from the positive control. Comparisons in the proximate analysis, amino acid profile and oligosaccharide concentrations of the unfermented and fermented samples were performed using one-way ANOVA followed by a Tukey post-test; observations were made in triplicate with one observation obtained daily. The assumption of no day effect was believed to be true. Data was expressed as mean ± SEM and considered to be significant if *P* < 0.05.

## Results

### Isolates with high protease, cellulase and amylase activities

**Fig. 1:**
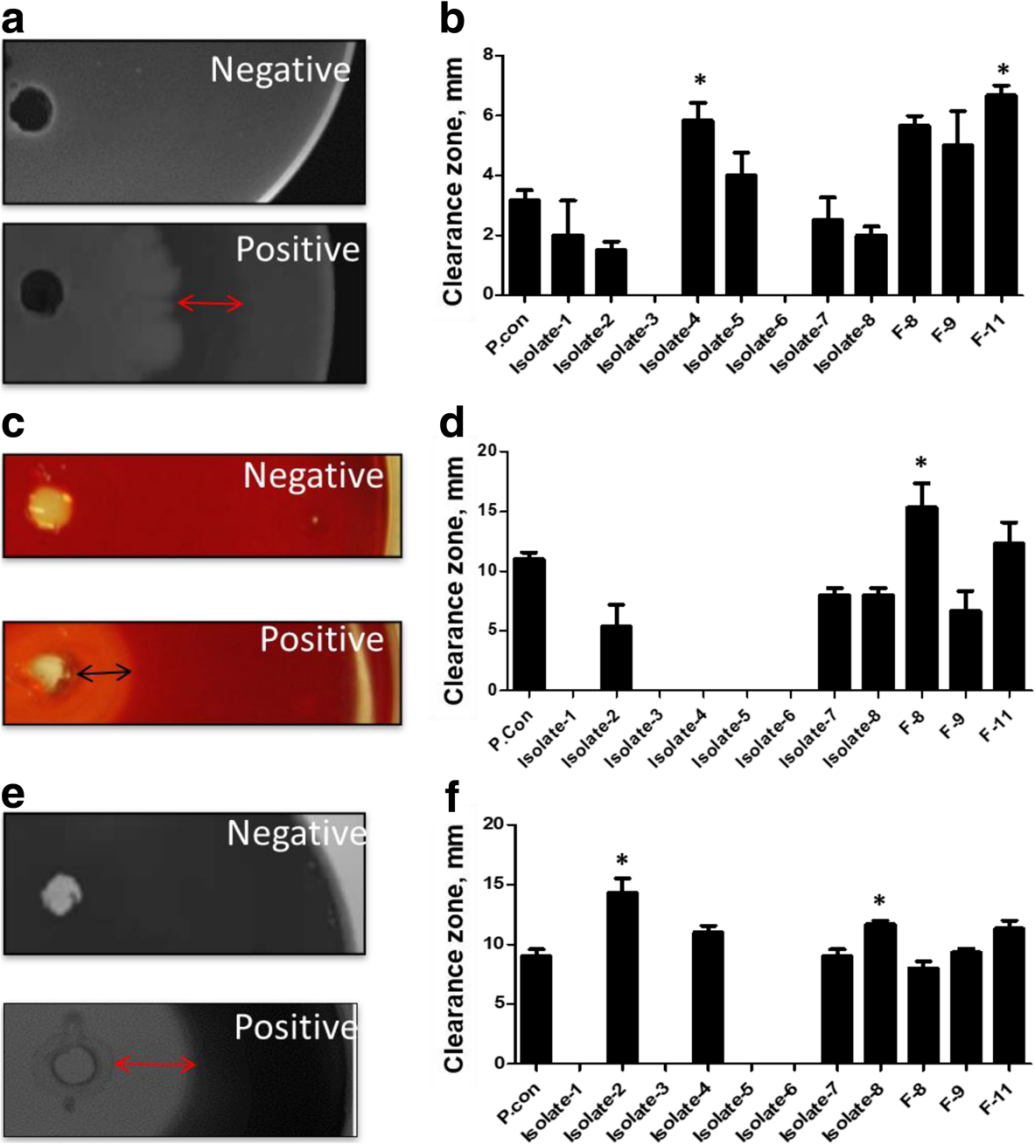
Representative images and clearance zone measurements indicate proteinase (**a**, **b**), cellulase (**c**, **d**), and amylase (**e**, **f**) activities of isolates derived from fermented soybean foods or from the grass carp intestine after 72 h incubation. The radius of each clearance zone, reflecting the enzyme activity, were measured. Error bars represent the SEM of the means data of three independent observations. *indicates significant difference compared to the unfermented soybean meal control (*P <* 0.05)

All bacteria isolates demonstrated protease activity on the soymilk agar plates and the isolate having the largest clearance zone was Isolate-4 from food origin and Isolate 11 from fish intestine. Protease activity of Isolate-4 and F-11 was greater than that of *B. subtilis* ATCC6633 (positive control; *P <* 0.05; Fig. 1a). All three isolates derived from fish intestine were tested positive for cellulase activity and only Isolate-2, Isolate-7 and Isolate-8 of food origin also demonstrated cellulase activity (Fig. 1b). Of all the isolates tested only F-8 had a greater cellulase activity (*P <* 0.05; Fig. 1d) when compared to the positive control (Fig. 1c). Food-origin isolates, Isolate-2, Isolate-4, Isolate-7 and Isolate-8, and the three fish-origin bacteria also tested positive for amylase activity. The Isolate-2 and Isolate-8 demonstrated significantly higher amylase activity (*P <* 0.05; Fig. 1f) than that of the positive control (*P <* 0.05; Fig. 1e). A summary of the enzymatic activities of all bacteria isolated are shown in Table 2. A total of six isolates (Isolate-2, Isolate-7, Isolate-8, F-8, F-9 and F-11) were shown to have protease, cellulase and amylase activities and were further included in the solid-state fermentation analysis.

**Table 2:** Amino acid profile of soybean meal fermented for 24 h with different strains of bacteria isolates

	Unfermented	Isolate-2	F-9
Lys	2.8 ± 0.1^b^	3.24 ± 0.04^a^	3.21 ± 0.02^a^
Met	0.47 ± 0.01^a^	0.5 ± 0.03^a^	0.29 ± 0.01^b^
Thr	1.77 ± 0.06	1.83 ± 0.03	1.85 ± 0.03
Val	1.99 ± 0.06^b^	2.3 ± 0.07^a^	2.41 ± 0.03^a^
Ile	1.87 ± 0.09	2.09 ± 0.07	2.05 ± 0.05
Leu	3.41 ± 0.13	3.75 ± 0.05	3.56 ± 0.04
Arg	3.17 ± 0.11	3.10 ± 0.1	3.17 ± 0.11
His	1.18 ± 0.04^b^	1.41 ± 0.03^a^	1.43 ± 0.02^a^
Phe	2.19 ± 0.07^b^	2.57 ± 0.03^a^	2.57 ± 0.04^a^
Cys	0.57 ± 0.01^b^	0.65 ± 0.01^a^	0.36 ± 0.01^c^
Pro	2.22 ± 0.09^c^	2.53 ± 0.02^b^	5.11 ± 0.05^a^
Tyr	1.74 ± 0.04^b^	1.99 ± 0.02^a^	1.89 ± 0.03^a^
Asp	4.96 ± 0.17^b^	5.49 ± 0.03^a^	5.49 ± 0.03^a^
Ser	2.25 ± 0.08	2.24 ± 0.05	2.21 ± 0.04
Glu	8.36 ± 0.28^b^	10.42 ± 0.2^a^	9.74 ± 0.16^a^
Gly	1.92 ± 0.07^b^	2.12 ± 0.02^a^	2.11 ± 0.01^a^
Ala	1.93 ± 0.07	2.03 ± 0.04	1.93 ± 0.03
Total	42.80 ± 1.38^b^	48.26 ± 0.17^a^	49.38 ± 0.15^a^
EAAs	18.86 ± 0.62^b^	20.80 ± 0.16^a^	20.54 ± 0.17^a^
NEAA	23.94 ± 0.78^b^	27.46 ± 0.75^a^	28.85 ± 0.12^a^

Different superscript letters indicate significate differences at *P* < 0.05 in the row

### Characterization and identification of screened isolates

Isolates with tested enzymatic activities were identified as *Bacillus* using the MALDI-TOF MS (Table 2). Isolate-1 from black fermented beans and Isolate-5 from soft fermented beans were identified as *B. cereus*, and Isolate-7 and Isolate-8 from Japanese Natto were *B. subtilis*. Isolate-2 from red fermented beans, Isolate-4 from yellow fermented beans as well as F8, F9 and F11 of the fish-origin were unable to be identified to species level but gram staining identified the bacteria as gram positive bacilli.

By sequencing the partial 16S rRNA and *gyrB* genes, Isolate-2, Isolate-4, F-9, and F-11 were identified as *B. amyloliquefaciens*. The *gyrB* gene sequences of Isolate-2, Isolate-4, F-9 and F-11 had an identity of 98%, 95%, 98% and 97% with *B. amyloliquefaciens* subsp. *plantarum* strain FZB42 (GeneBank accession # NC_009725.1), respectively. The partial 16S rRNA gene of Isolate-2 and F-9 had a 99% identity with *B. amyloliquefaciens* strain IHB B 3373 (GeneBank accession # KF475869.1). The partial 16S rRNA sequences of Isolate-4 were 99% identical to *B. amyloliquefaciens* strain BAB-68 16S (GeneBank accession # KC250107.1) and F-11 shared 99% identity with *B. amyloliquefaciens* subsp. *Plantarum* UCMB5033 (GeneBank accession # HG328253.1). The F-8 was identified as *B. subtilis*. Its partial 16S rRNA gene sequence had a 99% identity to *B. subtilis* strain IHB B 4270 (GeneBank accession # KF475879.1) and its *gyrB* gene sequence had a 97% identity to *B. subtilis* subsp. *subtilis* str. 168 (GeneBank accession # NC_000964.3).

### Influences of solid-state fermentation on total soluble protein profiles

**Fig. 2:**
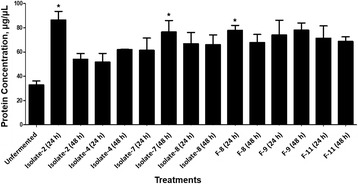
Concentration of total soluble peptides in soybean meal when fermented with different bacterial isolates at 24 and 48 h. The total soluble protein was quantified using the DC Assay. Error bars represent the SEM of the means data of three independent observations. *indicates significant difference compared to the unfermented soybean meal control (*P <* 0.05)

**Fig. 3:**
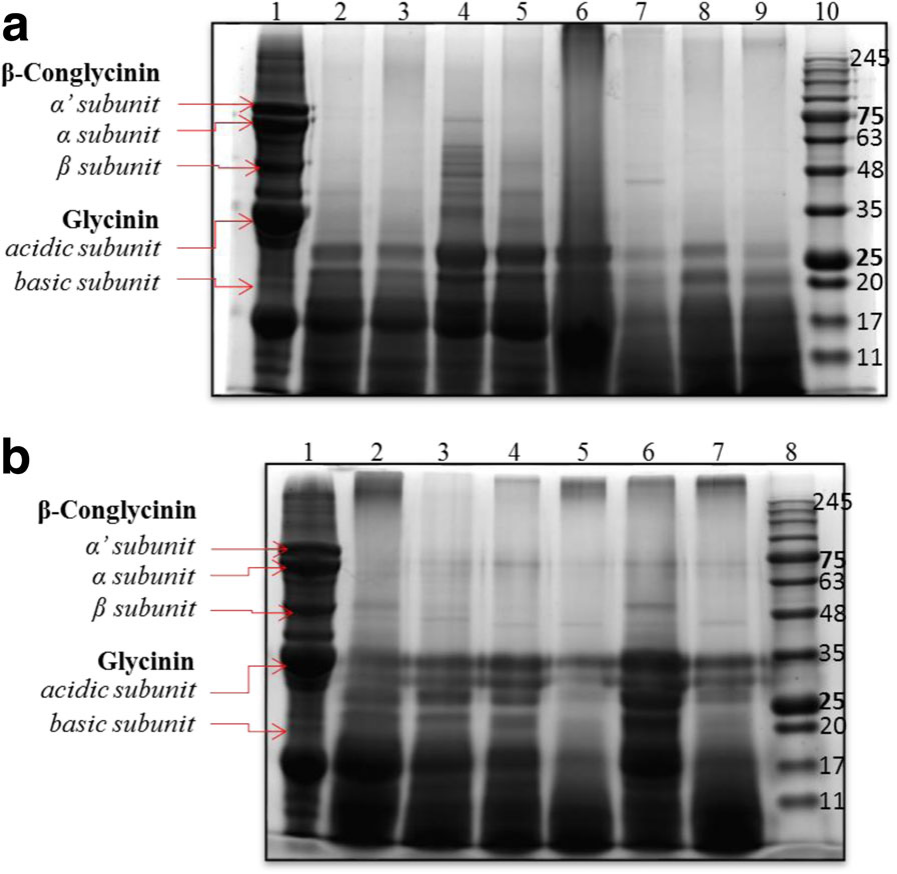
Total soluble peptide profile of soybean meal when fermented with different bacterial isolates at 24 and 48 h. **a** Bacteria isolated from fermented food sources. **b** Bacteria isolated from grass carp fish. Coomassie Blue staining representing the peptide profile. In Panel **a**, lane 1 = unfermented soybean meal, lane 2 = Isolate-8 at 24 h, lane 3 = Isolate-8 at 48 h, lane 4 = Isolate-7 at 24 h, lane 5 = Isolate-7 at 48 h, lane 6 = Isolate-2 at 24 h, lane 7 = Isolate-2 at 48 h, lane 8 = Isolate-4 at 24 h, lane 9 = Isolate-4 at 48 h, lane 10 = marker. In Panel **b**, lane 1 = unfermented soybean meal, lane 2 = F-8 at 24 h, lane 3 = F-8 at 48 h, lane 4 = F-9 at 24 h, lane 5 = F-9 at 48 h, lane 6 = F-11 at 24 h, lane 7 = F-11 at 48 h, lane 10 = marker

Total soluble protein concentrations in the fermented soybean meal were significantly increased by Isolate-2 and F-8 after 24 h solid-state fermentation and increased by Isolate-7 and F-9 after 48 h treatments when compared to the unfermented SBM control (Fig. 2). To analyze the influence of fermentation on the soybean meal protein profile, sodium dodecyl sulfate polyacrylamide gel electrophoresis was performed. The maximal degradation of large proteins in the soybean meal was almost complete at 24 h of fermentation (Fig. 3), however, the distributional pattern was different between food-origin and fish-origin isolates. As shown in Fig. 3, the fermentations using both the food and fish intestine sourced bacteria were successful in reducing the antigenic protein β-conglycinin including the α’-subunit (76 kDa), α-subunit (72 kDa) and β-subunit (53 kDa). The food sourced bacteria (Fig. 3, lanes 2–9) fermentation can degrade most of the soybean meal soluble proteins to 25 kDa (Fig. 3a), which was approximately 10 kDa smaller than the proteins fermented by bacteria of fish origin (Fig. 3b; lanes 2–7).

**Fig. 4:**
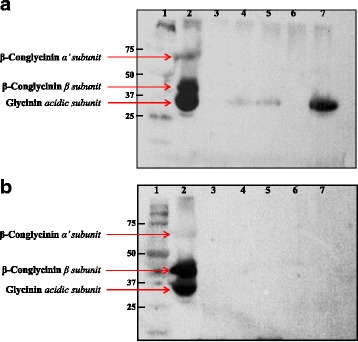
Western blot indicating piglet immunoreactivity to the fermented soybean meal products. **a** Soybean meal fermented with various isolates for 24 h. **b** Soybean meal fermented with various isolates for 48 h. Lane 1 = marker, lane 2 = unfermented soybean, lane 3 = Isolate-2, lane 4 = Isolate-4, lane 5 = F-8, lane 6 = F-9, lane 7 = F-11

Western blot analysis using the serum of soybean meal-challenged piglets as the primary antibody demonstrated immunoreactivities at approximately 75 kDa, 40–50 kDa, and 30–40 kDa for the unfermented soybean meal (Fig. 4). At 24 h of fermentation, immunoreactivities were abolished in fermented soybean meals treated with Isolate-2 and F-9 (Fig. 4a, lanes 3, 6) although reduced immunoreactivity was still present in the soybean meals treated with Isolate-4, F8 or F11 (Fig. 4a, lanes 4, 5, and 7, vs. unfermented soybean meal in lane 2). By 48 h, no immunoreactivity was visible in all fermented products (Fig. 4b).

### Changes in crude protein and amino acids profiles after fermentation

Crude protein analysis showed that solid-state fermentation significantly increased crude protein concentrations from 48.05 ± 0.47% to 53.37 ± 0.67% with Isolate-2 or 56.32 ± 0.73% with F-9 at 24 h, respectively. As shown in Table 2, the total amino acids as well as essential (EAAs) and nonessential amino acids (NEAAs) were also increased after 24 h-fermentation with Isolate-2 and F-9. The soybean meal fermented with Isolate-2 was greater in lysine, valine, histidine, phenylalanine, cysteine, proline, tyrosine, asparagine, glutamine and glycine when compared to the unfermented control. With F-9, lysine, valine, histidine, phenylalanine, cysteine, proline, tyrosine, asparagine, glutamine and glycine were increased, but sulfur-containing amino acids methionine and cysteine were decreased.

### Oligosaccharides

The unfermented soybean meal contained 9.88 ± 0.20 mg/g raffinose and 41.73 ± 1.70 mg/g stachyose; at 24 h of fermentation, both oligosaccharides levels were below the detection levels in the soybean meal.

## Discussion

From fermented soy foods and the grass carp intestine six isolates of *Bacillus* species were identified to have protease, cellulase and amylase activities. It was consistent with previous studies that the isolated bacteria from other traditionally fermented foods, such as Dawadawa [31], Meju [25], Cheonggukjang [43], and Douchi [44], were *Bacillus* species. These bacteria can survive on legumes because they secrete extracellular enzymes, such as proteases [25]. Among the isolates, Isolate-2 of food-origin and F-9 of fish-origin were identified as the most efficient isolates, which eliminated most of the antigenicity againstβ-conglycinin and glycinin and achieved the maximal soluble protein fractions, crude protein as well as amino acids profiles at 24 h.

Isolate-2 and F-9 were further identified as *B. amyloliquefaciens* by sequencing 16S rRNA and *gyrB* genes. The *gyrB* gene is responsible for producing the β subunit protein of a DNA gyrase and is essential in DNA replication [45]. This gene is distributed universally among species and has a faster rate of molecular evolution than the 16S rRNA gene and therefore is more accurate in identification of bacteria strains [45]. In general, the results using the 16S rRNA gene were consistent with the *gyrB* gene. One of the isolates F-11 was identified as *B. subtilis* using the partial 16S rRNA while it was identified as *B. amyloliquefaciens* using the *gyrB* gene. *B. amyloloiquefaciens* has been less investigated in fermentation of soybean compared to *B. subtilis*. A recent study has used a *B. amyloliquefaciens* isolated from the Korean traditional soybean paste in the soybean meal fermentation and found it significantly improved the nutritional quality and bioactivity [46].

Solid-state fermentation was used in the present study because this application is more desirable to the agricultural industry setting due to less costly equipment and lower moisture levels in the liquid fermentation [47, 48]. Our results showed that solid-state fermentation using Isolate-2 and F-9 could degrade large proteins to less than 25 kDa or 35 kDa, respectively, and antigenicity against β-conglycinin and glycinin were completely eliminated in the soybean meal by 48 h of fermentation. A previous study using *B. amyloliquefaciens* isolated from soybean paste also demonstrated a reduction in proteins size to 27 kDa and under [46]. The reduction of protein sizes was important to increase the digestibility of soybean proteins [49]. Moreover, removal of these antigenic soybean proteins is beneficial for both digestion and animal health. Antigenic proteins found in soybean meal could activate the immune system leading to inflammation of the intestine and the release of compounds such as histamine, which increases the incidence of diarrhea [13, 16]. It is interesting that the degrading effect of food-origin *B. amyloliquefaciens* was quite similar, but displayed a distinct digested soybean meal protein profile when compared with that of the fish-origin bacteria isolates. It is possible that different species and subspecies of bacteria may secrete distinct arrays of proteinases that target different subsets of the soybean proteins.

Fermentations were also able to decrease the oligosaccharides and RFOs to non-detectable levels at 24 h. Previous studies have reported a decrease in the soy oligosaccharides, stachyose and raffinose, and NSPs when soybean meal was fermented with bacteria [50], although a much longer fermentation (10 d) was required. The reduction of these oligosaccharides could have been due to hydrolysis of the α-1,6-galactose linkage by the enzyme α-1,6-galactosidase. Some strains of *Bacillus* have been found to secrete extracellular α-1,6-galactose [51] which may explain the reduction of oligosaccharides observed. Further characterization of the extracellular enzymes secreted by Isolate-2 and F-9 should be investigated to support the observation.

Fermentation could substantially increase the crude protein contents of the soybean meal [24], however, the efficacy could vary among different microorganisms. In the present study, the crude protein was significantly increased by 5.32% and 8.27% following 24 h of fermentation with the two *B. amyloliquefaciens* isolated from fermented food and fish intestine, respectively. The fermentation with *B. amyloliquefaciens* derived from soybean paste was found to increase the crude protein content by 6.42% [46]. In the same study, fermentation with two *Lactobacillus* spp. and *Saccharomyces cerevisiae* was observed to increase the crude protein by 1.05%, 1.91% and 5.61%. Soybean meal fermented with *Aspergillus oryzae* has been reported to have a crude protein increase ranging from 1.95% [52] to 10% [20]. The increase in crude protein may be explained by two processes. Firstly, the increased crude protein observed could be due in part to the decrease in carbohydrates [20]. Some bacteria can break down cellulose, polysaccharides, and oligosaccharides and utilize its sugar subunits for their metabolism and respiration processes [48].

In addition to the crude protein, the amino acids profiles of the fermented soybean meal were improved with Isolate-2 and F-9. The total amino acids were increased by 13% and 15% with the amino acid profiles sharing a similar pattern between the soybean meals treated with Isolate-2 and F-9, respectively. The EAAs, including lysine, valine, histidine and phenylalanine were increased in the fermented soybean meal from a range of 15–19%. There existed differences in amino acids between the soybean meal fermented with Isolate-2 and that fermented with F-9. The proline content was greater while the methionine and cysteine amino acids being lower in samples treated with F-9 when compared to Isolate-2 and the unfermented control. The alterations in amino acid profiles had a great relationship with the microorganism used. Methionine rather than lysine was increased in the soybean meal fermented with *B. amyloliquefaciens* [46]. Hong et al. observed a significant increase in glycine, glutamic acid, and aspartic acid when soybean meal was fermented with *A. oryzae* [20]. Using cracked soybean fermented with *A. oryzae* it was reported that there was a significant increase in all amino acids except arginine and lysine [53]; by contrast, cracked soybeans fermented with *B. subtilis* increased aspartic acid, glutamic acid, serine, alanine, proline, valine, methionine, cysteine, isoleucine, phenylalanine, tyrosine and lysine levels after 48 h [53]. The current study, unlike some of the previous ones was able to increase lysine and threonine, two limiting amino acids of the corn-soybean meal for swine, which could decrease the requirement of synthetic amino acids to be added.

Fermented soybean meal has also been shown to improve animal performance. A previous study performed using the spores of *B. amyloliquefaciens* to ferment whole soybean as a target dietary supplement for use in poultry feeds was able to increase growth rate and feed efficiency by 8% and 8.8%, respectively [54]. Also, this species of bacteria has been found to have anti-bacteria activity [55] and antioxidative activity [46, 55], which may be able to improve growth performance for weaned piglets. The effect of replacing some soybean meal using soybean meal fermented with the two *B. amyloliquefaciens* strains isolated in the current study on animal production will be investigated in future studies.

## Conclusions

In conclusion, fermentation using the newly isolated food-derived Isolate-2 and fish-derived F-9 resulted in an improved protein profile, an increase in crude protein concentration, an increase in total amino acid concentration, and a decrease in raffinose and stachyose in the soybean meal. These two natural derived bacteria have the advantages of being safe, time-efficient and good at producing a more balanced amino acid profile. They could be good seeding bacteria in the solid state fermentation of soybean meal with the goal of improving the health of weaned piglets.

## Abbreviations

BHI: Bacto Brain Heart Infusion
CMC: Carboxymethyl cellulose
EAA: Essential amino acids
LB: Luria Bertani
MALDI-TOF MS: Matrix assisted laser desorption ionization- time of flight mass spectrometry
MRS: DeMan, Rogosa and Sharpe
NSP: Non-starch polysaccharides
RFO: Raffinose family oligosaccharides
SDS-PAGE: Sodium–dodecyl sulfate-polyacrylamide gel electrophoresis
